# Internet Use and Epidemiologic Investigation of Gastroenteritis Outbreak

**DOI:** 10.3201/eid1003.020607

**Published:** 2004-03

**Authors:** Markku Kuusi, J. Pekka Nuorti, Leena Maunula, Ilkka Miettinen, Hannu Pesonen, Carl-Henrik von Bonsdorff

**Affiliations:** * National Public Health Institute, Helsinki, Finland; † Helsinki University Central Hospital Laboratory Diagnostics, Helsinki, Finland; ‡ National Public Health Institute, Kuopio, Finland; § Environmental Health Unit, Nurmes, Finland; ¶ Helsinki University, Helsinki, Finland

**Keywords:** Internet, Outbreak, Survey, Norovirus, Water supply

In March 2000, a large outbreak of gastroenteritis occurred in a community where a regional computer network provides free Internet access for 42% of the households. We conducted an epidemiologic investigation using the Internet for data collection. Norovirus was identified in stool samples of nine patients but not in the municipal water supply. Of households with access to the network, 19% participated in the survey. The overall attack rate by household was 63%. Drinking water from the nonchlorinated community water system was associated with illness (relative risk [RR] 1.6; 95% confidence interval [CI] 1.1 to 2.2); drinking water only from a private well was associated with decreased likelihood of illness (RR 0.3; 95% CI 0.1 to 0.8). Data collection through the Internet was efficient. Internet surveys may become more common in epidemiologic investigations and have the potential to provide data rapidly, enabling appropriate public health action. However, methods should be developed to increase response rates and minimize bias.

The Internet is increasingly influencing the practice of epidemiology. It has been used for disseminating health information, providing access to journals, and managing multicenter randomized controlled trials (*1*). During an investigation of a syphilis outbreak (*2*), the Internet was used to notify patients’ sexual partners and to increase community awareness. Use of the Internet has also been evaluated as a risk factor for sexually transmitted diseases (*3*). The Internet provides advantages in data collection and collation, which can reduce the resources and workload required for questionnaire studies (*4*). However, there are no reports of community-based outbreak investigations conducted by using the Internet.

Noroviruses are common etiologic agents of epidemic gastroenteritis. In March 2000, a widespread norovirus outbreak occurred in a municipality in eastern Finland. We conducted a community-based epidemiologic investigation to determine the magnitude and source of the outbreak. A community computer network and the Internet were used for data collection. We assessed the feasibility and methodologic aspects of Internet-based surveys in an outbreak investigation, including representativeness of the population sample and potential sources of bias.

## Material and Methods

### Setting and Description of Computer Network

Ylä-Karjala region comprises municipalities A (population 10,000), B (population 6,700), and C (population 3,000) in North Karelia in eastern Finland. In 1998, an experimental pilot project in the Finnish information society called Learning Ylä-Karjala was begun. The project aims to reduce high unemployment and outmigration by providing a community computer network with free access to the Internet, local discussion groups, local information areas, and email for private citizens, state and municipal authorities, private companies, and other organizations. Approximately 3,500 households (42% of households in the municipalities) with 4,100 persons regularly use the regional network. At the end of 1999, 21% of the residents in municipality A, 20% in municipality B, and 22% in municipality C were registered users of the network. Persons who do not own a computer can access the network from public computers located in libraries, shops, and cafés. Most users (92%), however, access the network from a home computer. Each week, approximately 2,500 registered users log on to the network; 25% of registered users log on daily.

### The Outbreak

On March 10, 2000, the National Public Health Institute was notified of an outbreak of gastroenteritis in municipality A. During the next 2 weeks, an increasing number of patients with vomiting, diarrhea, or both were identified throughout the municipality. The clinical features were typical of norovirus gastroenteritis: 1–2 days’ duration and vomiting and nausea as predominant symptoms. On March 24, small spherical viruses suggestive of norovirus were detected in stool specimens of patients by electron microscopy. Wide distribution of cases in municipality A and outbreaks among groups of tourists visiting that municipality who reported drinking municipal tap water suggested that this municipality’s water supply may have been contaminated. No increases in incidence of gastroenteritis were reported in neighboring municipalities B and C.

Municipality A’s water system provides groundwater to 75% of the population; the rest have private wells. As the water is not routinely chlorinated, the environmental health unit issued a boil-water notice on March 24, and the municipal water works started chlorinating the water. Municipalities B and C have their own, separate water systems.

### Epidemiologic Investigation

To investigate the outbreak, we conducted a survey among users of the community computer network in municipalities A, B, and C. Data were collected by using a standard online questionnaire, which is based on FirstClass software (Centrinity Inc., Richmond Hill, Ontario), posted on the network. Only persons who completed the questionnaire from a home computer were included in the study. The questionnaire was placed in a specific message area, and data from the completed questionnaire were transferred directly to a database. Data were transmitted to the server encrypted, and the server was protected by a firewall, both features included in FirstClass software. From April 10 to 24, when a user logged on to the network, a notice appeared on their screen requesting the user to complete the online questionnaire concerning the outbreak of gastroenteritis. To increase the participation rate, the survey was advertised on two different days in the local newspaper.

A case-patient was defined as member of a household with at least one registered user of the community computer network who had an episode of diarrhea (>3 loose stools/day), vomiting, or both during March 2000. If more than one resident of the household was ill, only the one with first onset of illness was requested to complete the questionnaire. Participants were asked about symptoms, onset of illness, and exposures to drinking water from different sources during March 1 to 31, 2000.

Data were analyzed by using EpiInfo version 6.04 (Centers for Disease Control and Prevention, Atlanta, GA). We calculated attack rates (AR) by municipality and household as well as the relative risks (RR) and 95% confidence intervals (CI) for drinking any water from the municipal water supply, unboiled water from the supply, and well water.

### Laboratory and Environmental Investigations

Stool specimens from 23 patients who were treated for acute gastroenteritis at the community health center of municipality A during March 21 to 31 were examined for viruses by electron microscopy and by the reverse transcription–polymerase chain reaction (RT-PCR) test for Norovirus, Sapovirus, and Astrovirus (*5*,*6*). Routine bacterial cultures and microscopy for parasites were also performed for the specimens. Nine water samples from the water supply of municipality A were investigated for coliforms and noroviruses, as described previously (*7*).

## Results

### Epidemiologic Investigation

A total of 672 persons in the three municipalities completed the online questionnaire. These persons represented an estimated 19% of registered households with access to the network. Of all respondents, 508 (76%) were from municipality A, 59 (9%) from municipality B, and 51 (8%) from municipality C. Forty-one percent of respondents were men, and 59% women. The median age was 27 years (range 6–74). In municipality A, the demographic characteristics of respondents were different from the population of the municipality: the proportion of young adults 15–28 years of age was higher, and the proportion of persons >65 years of age was much lower than in the general population (Table 1).

**Table 1:** Attack rates of gastroenteritis by age group in municipality A, eastern Finland, March 2000

Age (y)	Population (%)	Respondents (%)	Cases	AR (%)^a^
0–14	1,743 (17)	72 (14)	43	60
15–28	1,479 (15)	183 (36)	123	67
29–64	4,975 (49)	249 (49)	152	61
>65	1,911 (19)	4 (1)	1	25
Total	10,108 (100)	508 (100)	319	63

^a^AR, attack rate; based on first episode of illness occurring in the household.

Of respondents, 368 (55%) met the case definition; 60% were female. The median age was 27 years (range 8–70). The epidemic curve (Figure) shows a fluctuating outbreak with several peaks. Cases began to increase in early March; the incidence peaked during March 19 to 23. Of the municipalities, the attack rate by household was highest (63%) in municipality A compared with that in municipalities B (17%) and C (29%) (RR 2.8; 95% CI 2.0 to 3.9). By direct standardization of the age-specific attack rates to the population, the total number of ill in municipality A was 5,500. Among residents of that municipality, drinking any unboiled tap water was significantly associated with illness (AR 65% vs. 42%; RR 1.6; 95% CI 1.1 to 2.2), while drinking water from a private well was associated with decreased likelihood for illness (AR 24% vs. 71%; RR 0.3; 95% CI 0.1 to 0.8) (Table 2).

**Figure:**
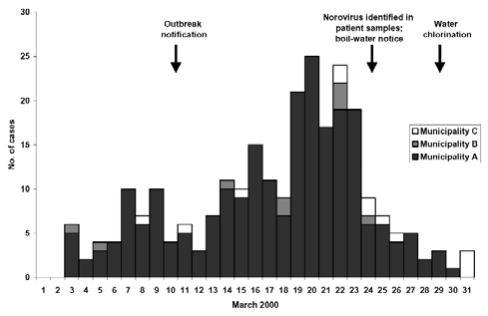
Cases of gastroenteritis by date of illness onset in a Norovirus outbreak, eastern Finland, March 2000. Based on first episode of illness occurring in the household.

**Table 2:** Factors associated with gastroenteritis among residents of municipality A, Finland^a^

	Exposed			Not exposed				
Risk factor	Ill	Total	AR (%)	Ill	Total	AR (%)	RR	95% CI
Any unboiled tap water	299	460	65	20	48	42	1.6	1.1 to 2.2
Unboiled tap water at home	251	366	69	19	54	35	2.0	1.4 to 2.8
Unboiled tap water outside home	240	347	69	50	112	45	1.6	1.3 to 1.9
Well water only	4	17	24	145	204	71	0.3	0.1 to 0.8

^a^AR, attack rate; RR, relative risk; CI, confidence interval.

### Laboratory and Environmental Investigations

Nine stool specimens (one from a survey participant) were positive for Norovirus by RT-PCR. In two additional patients, electron microscopy results for small, round spherical viruses were positive. All stool samples were negative for bacterial pathogens and parasites. All samples from the municipal water supply were negative for noroviruses by RT-PCR; the samples also were negative for indicator bacteria. Inspection of the water supply system did not indicate the site of contamination.

## Discussion

In investigating this widespread Norovirus outbreak, collecting data through the Internet was efficient and yielded a sample of the population with sufficient statistical power. Among residents of municipality A, drinking tap water was associated with illness, whereas drinking well water exclusively was associated with decreased likelihood of illness.

Widespread Norovirus outbreaks are common, and investigations may require substantial resources. In our study, electronic collection of data had two major advantages. First, when questionnaires were completed online instead of through a mail-in or telephone survey, several days were saved. Second, appropriate planning of the electronic questionnaire and direct transfer of data into a database saved much time and allowed a large sample size to be obtained without additional resources. However, Internet data collection may cause other delays. It takes time to set up the Web page and check its proper function. In our study population, only 25% of users logged on daily, and half of registered users did not log on during a whole week, while most people likely answer the phone and check their mail daily.

Our study had several limitations. Of the households in our study area, only 42% had access to the computer network. In telephone surveys, noncoverage is usually not a major concern (*8*). In community-based Internet surveys, noncoverage may be a substantial problem, depending on the topic studied. Because we only included persons who completed the questionnaire from a home computer, young people were overrepresented among respondents of our online survey. Also, although detailed demographics of registered users of the community network were not available, young age groups and persons with higher education and income are overrepresented among Internet users (*1*). Only four respondents were >65 years, although this group represents nearly 20% of the population. However, representativeness of participants may also be a problem in studies that use traditional methods for enrolling participants such as random digit dialing (*9*).

In our survey, 19% of households with access participated, but defining the exact sampling frame was not possible. This problem also is similar to telephone surveys using random digit dialing (*10*). In recent studies using that dialing system, estimated response rates from 28% to 35% have been reported (*9*,*11*). In an email study of a defined group of employees in Alaska, 91% of questionnaires were returned (*12*). Company employees likely have access to a computer and are also accustomed to checking email daily. The higher response rates in email studies are therefore not directly comparable with our survey. Whether response rates in studies that collect data electronically will ever be as high as in studies that collect information by telephone or mail is not known. In a recent investigation of an outbreak of conjunctivitis at a university, data were collected through email and the Internet, and the response rate was only 50% (*13*). Telephone or in-person surveys have higher participation rates and fewer missing data than surveys that use self-administered questionnaires (*14*).

The setting in our study was unusual because a single provider provided access to the Internet, which made it easy to distribute information about the survey to users. Most communities, however, are serviced by several Internet providers, making it more difficult to access users. Whether all commercial providers would be willing to interrupt the log-in procedure with this request and link to the outbreak investigation Web site is uncertain. As was done in our survey, advertising the study in newspapers and other media could be used to increase participation rates. Data access issues could also be problematic if the study were conducted through a commercial Internet provider.

We conducted searches in Medline, ScienceDirect, and ISI Web of Science (Available from: http://isi10.newisiknowledge.com), but we did not find published reports of community-based outbreak investigations in which data were collected through the Internet or through email. Internet surveys are likely to become more common in epidemiologic investigations and have the potential to rapidly provide data to enable appropriate public health action. In industrialized countries, most people will have access to the Internet and email within the next few years, providing epidemiologists increasing opportunities to conduct studies with online data collection. Defining the sampling frame and appropriate design of the questionnaire and database are essential. Response rates and demographics of respondents should be monitored to minimize selection bias. The method of choice for data collection in an outbreak investigation depends on the population and topic studied. Currently, online data collection seems best suited for investigations conducted in well-defined populations with high Internet coverage, where the exposures studied are unlikely to be strongly related to demographic and socioeconomic factors.
